# Cervical HSV-2 infection causes cervical remodeling and increases risk for ascending infection and preterm birth

**DOI:** 10.1371/journal.pone.0188645

**Published:** 2017-11-30

**Authors:** Devin McGee, Arianna Smith, Sharra Poncil, Amanda Patterson, Alison I. Bernstein, Karen Racicot

**Affiliations:** 1 Department of Obstetrics, Gynecology and Reproductive Biology, College of Human Medicine, Michigan State University, Grand Rapids, MI, United States of America; 2 Department of Translational Science and Molecular Medicine, College of Human Medicine, Grand Rapids, MI, United States of America; The Scripps Research Institute, UNITED STATES

## Abstract

Preterm birth (PTB), or birth before 37 weeks gestation, is the leading cause of neonatal mortality worldwide. Cervical viral infections have been established as risk factors for PTB in women, although the mechanism leading to increased risk is unknown. Using a mouse model of pregnancy, we determined that intra-vaginal HSV2 infection caused increased rates of preterm birth following an intra-vaginal bacterial infection. HSV2 infection resulted in histological changes in the cervix mimicking cervical ripening, including significant collagen remodeling and increased hyaluronic acid synthesis. Viral infection also caused aberrant expression of estrogen and progesterone receptor in the cervical epithelium. Further analysis using human ectocervical cells demonstrated a role for Src kinase in virus-mediated changes in estrogen receptor and hyaluronic acid expression. In conclusion, HSV2 affects proteins involved in tissue hormone responsiveness, causes significant changes reminiscent of premature cervical ripening, and increases risk of preterm birth. Studies such as this improve our chances of identifying clinical interventions in the future.

## Introduction

Preterm birth (PTB), or birth before 37 weeks gestation, affects approximately 12% of pregnancies in the United States[1–7] and is the leading cause of neonatal mortality worldwide[4, 6, 8]. Despite its frequency and numerous consequences, PTB rates have increased over the last 30 years[2, 5, 7]. Progress in improving PTB rates has been slow due to its complexity and the lack of understanding of the underlying causes of the condition. Indeed, PTB is better defined as a syndrome[2], and labor is the clinical outcome that results from an accumulation of risk factors or pathologies[9].

The best characterized risk factor for PTB is inflammation associated with bacterial infection. Despite years of research, we are still largely unable to predict or prevent even these cases of PTB[2, 10]. Viruses were relatively uncharacterized in the reproductive tract during pregnancy but, with improved technologies for detection, i.e. PCR, they have recently garnered more attention. Specifically, cervical viral infections with herpes simplex virus-2 (HSV2) and human papillomavirus are being established as risk factors for PTB in women[11–17]. A mouse model of systemic viral infection during pregnancy also showed that murine gammaherpesvirus-68 (MHV68) infection increased risk for PTB, specifically in the presence of bacterial endotoxin[18, 19]. Systemic MHV68 was also found to preferentially infect the pregnant cervix in a mouse model[20].

Throughout most of pregnancy, the uterine cervix serves as a structural and immune barrier that supports and protects the developing fetus[9, 21]. The stromal extra-cellular matrix (ECM) consists of a dense network of highly cross-linked collagen fibers, which provide the cervix with the mechanical strength needed to physically support the fetus. The cervical epithelial cells synthesize a highly effective mucus barrier that protects the fetus from ascending bacteria. At the end of gestation the cervix undergoes significant remodeling, collectively termed “cervical ripening”, resulting in complete structural reorganization of the cervix in preparation for labor[9, 21].

Cervical ripening is associated with the functional loss of progesterone (P_4_) signaling within cervical tissue[22, 23]. This is not a result of systemic decline in P_4_, but tissue-specific enzymatic conversion of active P_4_ to its metabolites[9, 22, 23]. Although there are few studies characterizing estradiol (E_2_) function at this time, it likely becomes functional within the cervix as local P_4_ declines and systemic E_2_ is increasing. Other changes at this time include increased hyaluronic acid (HA) synthesis[24], epithelial proliferation and changes in mucus composition. It is thought that these changes are necessary, at term, to ensure the cervix is prepared for vaginal delivery. Conversely, if these changes are premature they could result in cervical insufficiency, which is associated with ascending infection, intra-amniotic inflammation and PTB[25].

It is our hypothesis that viral infection affects the homeostasis of the cervix during pregnancy, and induces changes associated with cervical ripening prematurely. We propose these changes will, thus, reduce structural support of the developing fetus and affect the barrier to bacterial infections. To test this, we established a mouse model of sexually transmitted HSV2 infection to best mimic the clinical scenario in women. We then used this model to determine if viral infection induced changes associated with premature cervical ripening and determined if these changes increased risk of PTB associated with intra-vaginal bacterial infection.

## Methods

### Pregnant mice and infections

Animals were maintained at Michigan State University’s Animal Facility and all procedures are approved by Michigan State University’s Institutional Animal Care and Use Committee. C57BL/6 mice were purchased from Jackson Laboratory (Bar Harbor, ME); adult female mice (8–12 wks of age) were mated and confirmed pregnant when vaginal plug was detected. To study cervical phenotypes following viral infection, mice received HSV2 (10^5^), or vehicle, intra-vaginally, on GD10.5 after sedation with isoflurane. We previously confirmed this treatment resulted in HSV2 infection of the cervix with PCR (protocol under viral quantification) and we noted redness and swelling of the external genitalia in HSV2- treated mice. The genital tract was monitored daily following HSV2 infection and scored on a 5-point scale, to verify infection and ensure humane treatment of animals. Based on previously reported work, the scoring was as follows: 1, slight redness of the external vagina; 2, swelling and redness of the external vagina; 3, severe swelling and redness of both vagina and surrounding tissue and hair loss in genital area; 4, genital ulceration with severe redness, swelling, and hair loss of genital and surrounding tissue; 5, severe genital ulceration extending to surrounding tissue, as previously describe by Kaushic and associates[26]. Mice with scores of 4 or 5 were sacrificed although scores of 4/5 did not occur in any animals with HSV2 from GD10.5-GD15.5. Mice were humanely sacrificed on GD15.5 using CO_2_ followed by cervical dislocation. The cervix was flushed with sterile 1XPBS (3x with 30μl) and fixed in 4% PFA overnight. Animal numbers per group: Virus, n = 8; control, n = 8. To determine how HSV2 affected PTB associated with intra-vaginal infection, pregnant mice were infected with HSV2, intra-vaginally on GD10.5, with *E*. *coli* (serotype O55), intra-vaginally on GD16.5, or HSV2 (GD10.5) + E. coli (GD16.5). Mice were then monitored for preterm birth (birth of non-viable pups within 48h). Animal numbers per group: Virus only, n = 8; *E*. *coli* only, n = 6; Virus + *E*. *coli*, n = 10.

### Virus production and quantification

HSV2 was passaged in Vero Cells (CL-81, ATCC, Grand Island, NY) in DMEM plus 10% FBS. After lysis supernatants were harvested, filtered (0.45 μm pore) and titered by 10-fold serial dilutions on confluent monolayers. To detect viral titers in mice, DNA was extracted from the cervix using DNeasy blood and tissue kit (69504, Qiagen, Valencia, CA). 100ng total DNA was then assayed using primers specific for HSV2 (forward primer: 5’-GCT-CGA-GTG-CGA-AAA-AAC-GTT-3’, reverse primer: 5’-TGC-GGT-TGA-TAA-ACG-CGC-AGT-3’) and compared to a standard curve. Results reported as copies/100ng DNA. MHV68 growth and quantification was previously described[20].

### *E*. *coli* growth and quantification

Live *E*. *coli* (serotype O55; ATCC) were grown in nutrient agar, plated, and quantified by colony formation. Vaginal inoculation was 20 μl bacteria equal to 10^5^ CFU, as previously described to attain 30% preterm birth. The dose of bacteria was confirmed by using an aliquot of leftover bacteria to plate and repeat quantification of colony-forming units after overnight incubation, as described previously[24].

### Histology

Cervices were harvested from mice and immediately placed into 4% paraformaldehyde rocking at room temperature overnight. Tissues were then washed in 70% ethanol, placed into Thermo Scientific Excelsior ES tissue processor for routine processing, and embedded in paraffin wax. For immunohistochemical (IHC) analysis of estrogen receptor (ER)-alpha, progesterone receptor (PR) and Ki-67 proteins, 6μm sections underwent antigen retrieval by boiling in sodium citrate buffer and treatment with endogenous biotin inhibitor (X059030-2, Dako, Santa Clara, CA). Tissue sections were incubated with primary antibody (ER-alpha, MC20, Santa Cruz Biotechnology; PR, A0098, Dako; Ki-67, RB-9106-S1, ThermoScientific) in antibody diluent (S080981-2, Dako) overnight at 4C. The following day, tissue was incubated with secondary antibody (ab6720, 1:200, goat anti-rabbit IgG-biotinylated, Abcam, Cambridge, ME) for 1h, treated with an inhibitor of endogenous peroxidase (S200380-2, Dako), and developed with diaminobenzidine (DAB) substrate-chromogen (K346711-2, Dako). Negative controls underwent the same protocol but without primary antibody. For collagen staining, 10μm tissue sections were stained with picrosirius red (Polysciences Inc, Warrington, PA). Using a 40x objective and polarized light, a photomicrograph was taken of 3 sections, 10 micrographs per section, at least 20μm apart, per animal. The optical density (OD) of 10 non-overlapping sections of each photo was analyzed. Specifically, after photomicrographs were converted to gray scale, they were inverted and OD was calculated using a calibrated threshold and the Rodbard standard curve (NIH Image J software). In this analysis, areas of dark collagen staining have low OD values, and areas with light staining have high OD values. To account for cell density, the OD was divided by cell nuclei/area. Animal numbers per group: Virus, n = 8; control, n = 8.

### Cell culture and treatments

Immortalized human ectocervical cells (ECT1, CRL-2614, ATCC, Grand Island, NY) were cultured in keratinocyte serum free medium (17005–042, Gibco, Grand Island, NY) with bovine pituitary extract and hEGF supplementation as recommended by ATCC under 5% CO2 at 37°C. Cells were confirmed to be mycoplasma free (13100–01, Southern Biotechnology, Birmingham, Al). Inhibitor of Src was an inhibitor cocktail, SKI-1/PP1 (ab120839, ab120859, Abcam, Cambridge, ME) resuspended in DMSO (5μM). Cells were treated with inhibitor for 1h prior to HSV2 infection (10^5^ PFU). Supernatants and cells were collected 24 or 48h after HSV2 infection, as indicated. To infect ECT-1 cells with HSV2, cells were inoculated with HSV2 (10^5^) in 500 μl for 1h prior to addition of 2.5 mL media (protocol for 35mm plate). Results from cell culture experiments are representative of 4 independent experiments.

### RNA, cDNA synthesis and qPCR

RNA was extracted from ECT-1 cells using RNeasy RNA extraction kit (74104, Qiagen, Valencia, CA). RNA concentration and purity was analyzed using spectrophotometric analyses of 260/280 ratios with exclusions for samples that were below 1.7. For real-time quantitative analysis of mRNA, 1 μg of RNA was reverse transcribed for each sample using iScript cDNA synthesis kit (170–8891, Bio-Rad, Hercules, CA). The cDNA was diluted 1:20 in nuclease free water and 5μl was mixed with SsoAdvanced Universal SYBR green superscript (172–5270, Bio-Rad, Hercules, CA) and gene specific primers for ER-alpha (forward primer: 5’-GGC-CCC-AGC-TCC-TCC-TCA-T-3’, reverse primer: 5’-ACG-TTC-TTG-CAC-TTC-ATG-CTG-TA-3’) and GAPDH (forward primer: 5’-AGG-GCT-GCT-TTT-AAC-TCT-GGT-3’, reverse primer: 5’-CCC-CAC-TTG-ATT-TTG-GAG-GGA-3’). Samples were evaluated with the Applied Biosystems qPCR machine. Values were normalized to GAPDH and calculated using delta delta Ct method; delta delta Ct = delta ct treated- delta Ct control; results expressed as fold differences from controls.

### Hyaluronic acid ELISA

Hyaluronic acid concentration was assessed using ELISA (DHYAL0, R&D systems, Minneapolis, MN). ECT1-conditioned medium was diluted 1:40, cervical flushes were diluted 1:160, and samples were assayed according to manufacturer's protocol. Wavelength correction was used by subtracting 540 nm readings from all readings at 450 nm. The subtraction was used to correct for optical imperfections in the plate as recommended by manufacturer.

### Western blot analysis

Cells were lysed in M-PER Mammalian Protein Extraction Reagent buffer (78503, Pierce, Rockford, IL), with HALT protease inhibitor. Total protein concentrations were quantified using BCA assay (23227, Pierce, Rockford, IL). Twenty-five micrograms of total proteins were dissolved in 1X sample buffer, boiled for 5 minutes and separated on a 5–20% SDS-PAGE gel in 1X Tris-Glycine SDS running buffer (Novex, Carlsbad, CA) at a constant voltage of 125V for 2h. The proteins were transferred to PVDF membranes (0.45 μm, Novex, Carlsbad, CA) in an XCell II Blot module apparatus (Novex, Carlsbad, CA) at a constant 25 V for 2hrs. Non-fat milk (NFM) (5%) was used to block non-specific signals and immunoblotting was performed with a 1:1000 dilution of primary antibodies against total Src and phospho-Src-Tyr527 (2105, 32G6, Cell Signaling, Danvers, MA), ER-alpha (MC20, Santa Cruz Biotechnology, CA), or 1:10,000 dilution of beta-actin (ab16039, Abcam, Cambridge, ME) in 2% NFM at 4°C overnight. Membranes were washed in 1X PBST and a 1:10,000 dilution of goat anti-rabbit or goat anti-mouse IgG-horseradish peroxidase (HRP) conjugate (Cell Signaling, Danvers, Mass) was used as appropriate. Membranes were developed using HRP substrate (Amersham ECL Prime Detection Reagent; General Electric, Buckinghamshire, UK) and immunoreactive proteins were visualized using the Bio-Rad ChemiDoc XRS+ and Image Lab Software (Bio-rad).

### Statistical analysis

Differences were determined using analysis of variance (ANOVA) (multiple comparisons, Tukey’s test), or independent t-test functions of Graph pad inSTAT statistical software (La Jolla, CA). A p-value of ≤ 0.05 was considered significant, different letters denote significant differences. Data is presented as mean ± standard error of the mean (SEM).

## Results

### Herpes simplex virus-2 infection increases PTB associated with *E*. *coli* infection

**Fig 1:**
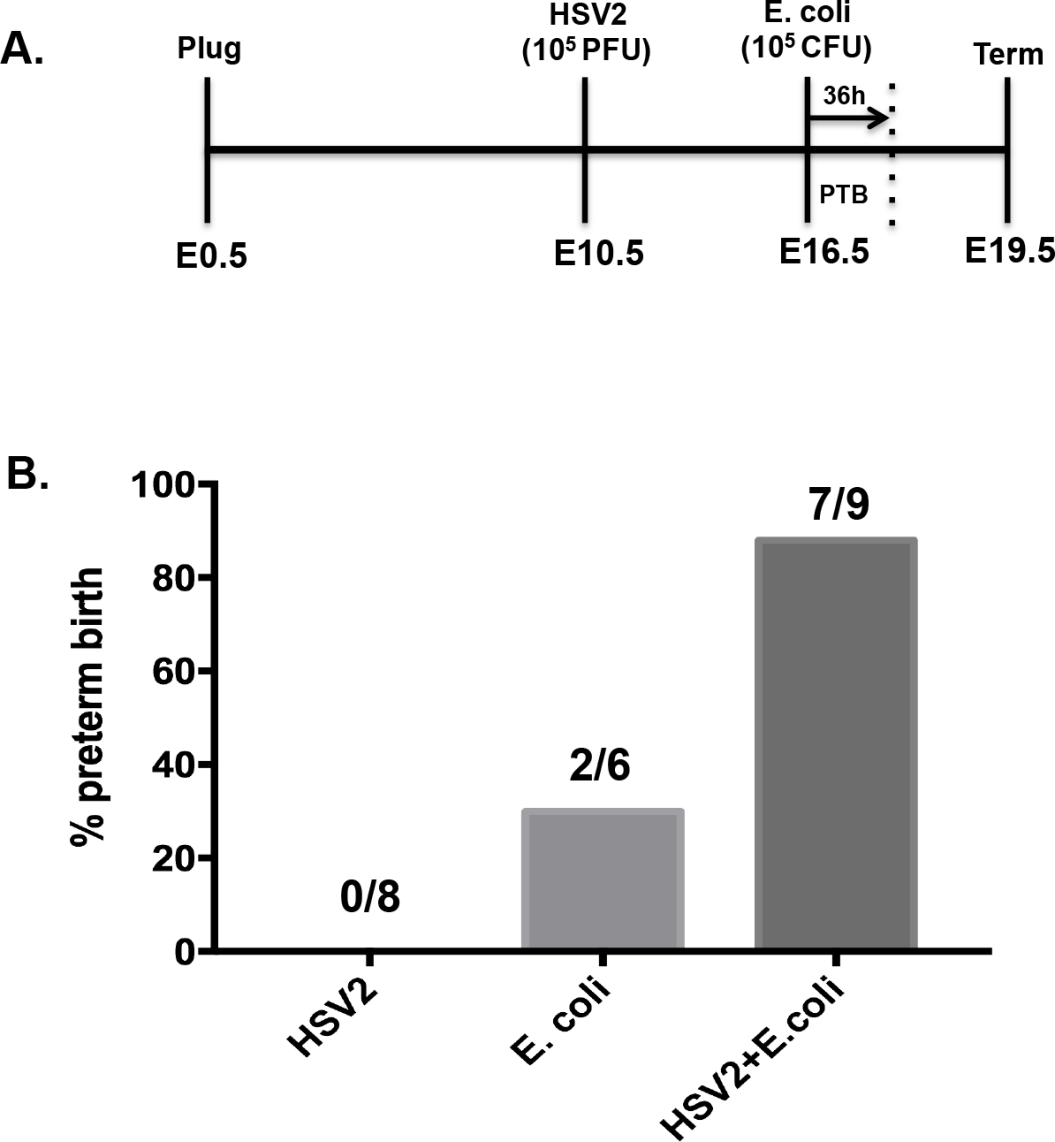
Herpes simplex Virus-2 infection increases PTB associated with intra-vaginal *E*. *coli*. (A) Mice were infected with HSV2 (10^5^ PFU) on gestational day (GD) 10.5, intra-vaginally, to mimic a sexually transmitted infection. At GD16.5 virus-infected and control mice were inoculated with pathogenic *E*. *coli* (10^5^ CFU), intra-vaginally, and rates of preterm birth were recorded. (B) Percentage of PTB for mice with HSV2 only, *E*. *coli* only, or HSV2 + *E*. *coli*. Number of animals depicted within graph.

A primary function of the pregnant cervix is to protect the upper reproductive tract from ascending infection. To determine if cervical HSV2 infection affected this function, we infected mice with HSV2 (10^5^ PFU) on gestational day (GD) 10.5, intra-vaginally, to mimic a sexually transmitted infection. At GD16.5 virus-infected and control mice were inoculated with pathogenic *E*. *coli* (10^5^ CFU), intra-vaginally, and rates of preterm birth were recorded (Fig 1A). Within 36h, 7/9 mice that received HSV2 and E. coli had PTB, 2/6 animals with E. coli alone had PTB, and 0/8 mice with HSV2 alone had litters that were preterm (defined as delivery of all pups within 36h of E. coli).

### Cervical viral infection affects stromal tissue organization

**Fig 2:**
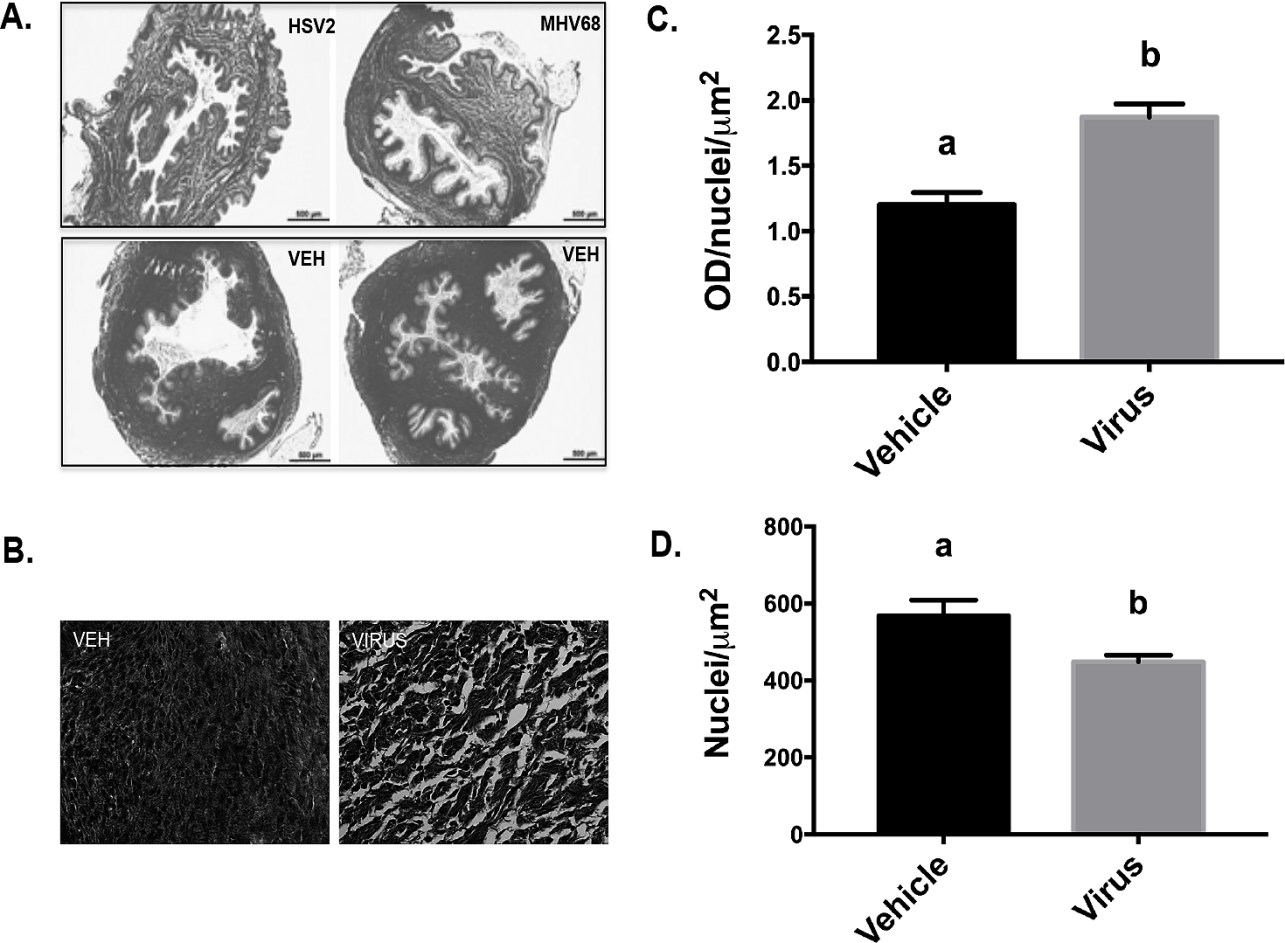
Cervical viral infection affects stromal tissue organization. Histological sections from cervices of animals infected with MHV68, HSV-2 or vehicle were stained with picrosirius red to characterize collagen organization. (A) Representative sections from pregnant mice with vehicle (Veh) or HSV2 were imaged under bright light microscopy (4x) and shown in gray scale. (B) Representative images from control (Veh) and infected (HSV2) animals under polarized light and gray scale conversion (40x). (C) Collagen content and structure was quantified by calculating the optical density (OD) using 3 non-overlapping photos from 10 sections per animal, normalized to cell nuclei/μm^2^. Specifically, after photomicrographs were converted to gray scale (as shown in (B)), they were inverted and OD was calculated using a calibrated threshold and the Rodbard standard curve. In this analysis, areas of dark collagen staining have low OD values, and areas with light staining have high OD values (a<b, t-test, p = .0007). (D) Cell density analysis, represented as the number of nuclei per μm^2^ (a>b, t-test, p = .02). Animal treatments: Vehicle, n = 8; Virus, n = 8.

Because ascending infections are often associated with structural changes in the cervix, like those that occur during cervical ripening, we next determined how cervical HSV2 infection affected the tissue organization of the cervix during pregnancy. We first determined if viral infection affected the cellular and/or extra-cellular structure of the cervical stroma. Mice were infected with HSV2 or vehicle, intra-vaginally, at GD10.5, and cervices were collected at GD15.5. Tissues were fixed, stained for collagen, and examined using bright light, and polarized light microscopy. Under bright light, it was readily apparent that infected cervices had a looser arrangement of collagen fibers compared to controls (Fig 2A). To quantify collagen objectively, the sections were first viewed under polarized light and converted to gray scale (Fig 2B). Images were then inverted and OD was calculated using a calibrated threshold and the Rodbard standard curve (NIH Image J). In this analysis, areas of dark collagen staining (dense collagen organization) are reported as low OD values, and areas with light staining (looser collagen organization) are reported as higher OD values, and the OD is normalized to cell number by dividing by number of nuclei per μm^2^. The infected cervix tissues had higher OD compared to the controls, indicating the collagen was less dense in infected tissue (Fig 2C), (a<b, t-test, p = .0007). The cervical stroma was also less cellular in infected animals, which was quantified by analyzing the number of nuclei per μ m^2^ (Fig 2D), (a>b, t-test, p = .02).

### Viral infection causes aberrant expression of estrogen receptor-alpha and progesterone receptor in cervical epithelial cells

**Fig 3:**
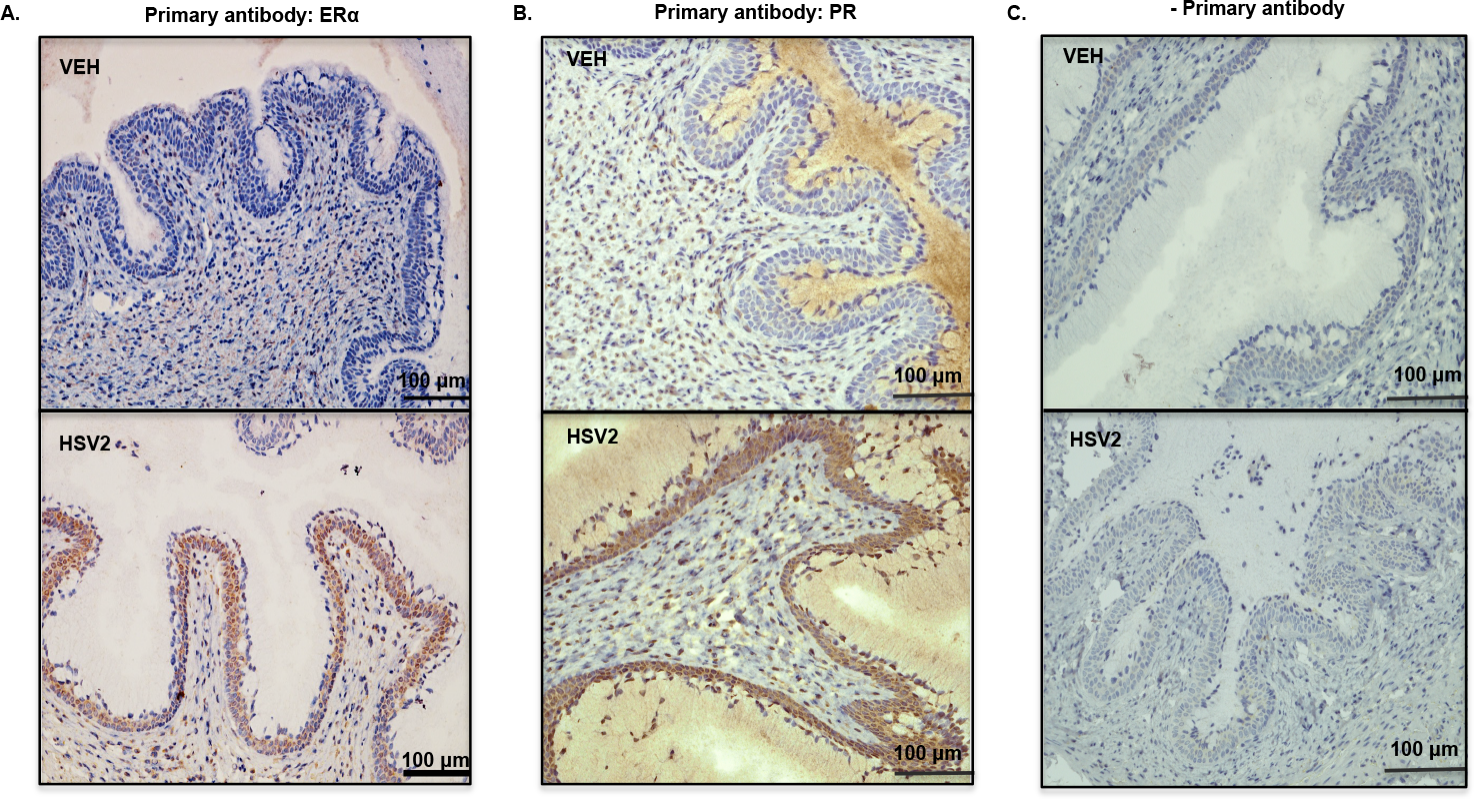
Viral infection causes aberrant expression of estrogen receptor and progesterone receptor in cervical epithelial cells. (A) Representative images from vehicle (VEH) and infected (HSV2) animals analyzed for ER-alpha or (B) PR protein using immunohistochemistry. (C) There was no staining in control assay lacking primary antibody. Animal treatments: Vehicle, n = 8; Virus, n = 8.

Cervical remodeling is associated with tissue-specific changes in steroid hormone signaling, characterized by a shift from progesterone to estrogen dominance[22, 23]. Therefore, we determined if HSV2 affected expression of progesterone receptor (PR) and estrogen receptor-alpha (ER-alpha), the primary mediators of hormone function. At GD15.5, vehicle-treated mice had very little, if any, ER-alpha staining in the cervical epithelium while HSV-infected mice had irregular pockets of ER-alpha staining (Fig 3A). Progesterone receptor was also absent in the epithelium of vehicle-treated mice at GD15.5, while it was highly expressed HSV2-infected mice (Fig 3B). There was PR staining in the cervical stroma of both vehicle and HSV2-infected mice at GD15.5 (Fig 3B). There was no positive staining in tissues when primary antibody was absent (Fig 3C).

### Epithelial cell proliferation and hyaluronic acid are increased by HSV2 infection of the pregnant cervix

**Fig 4:**
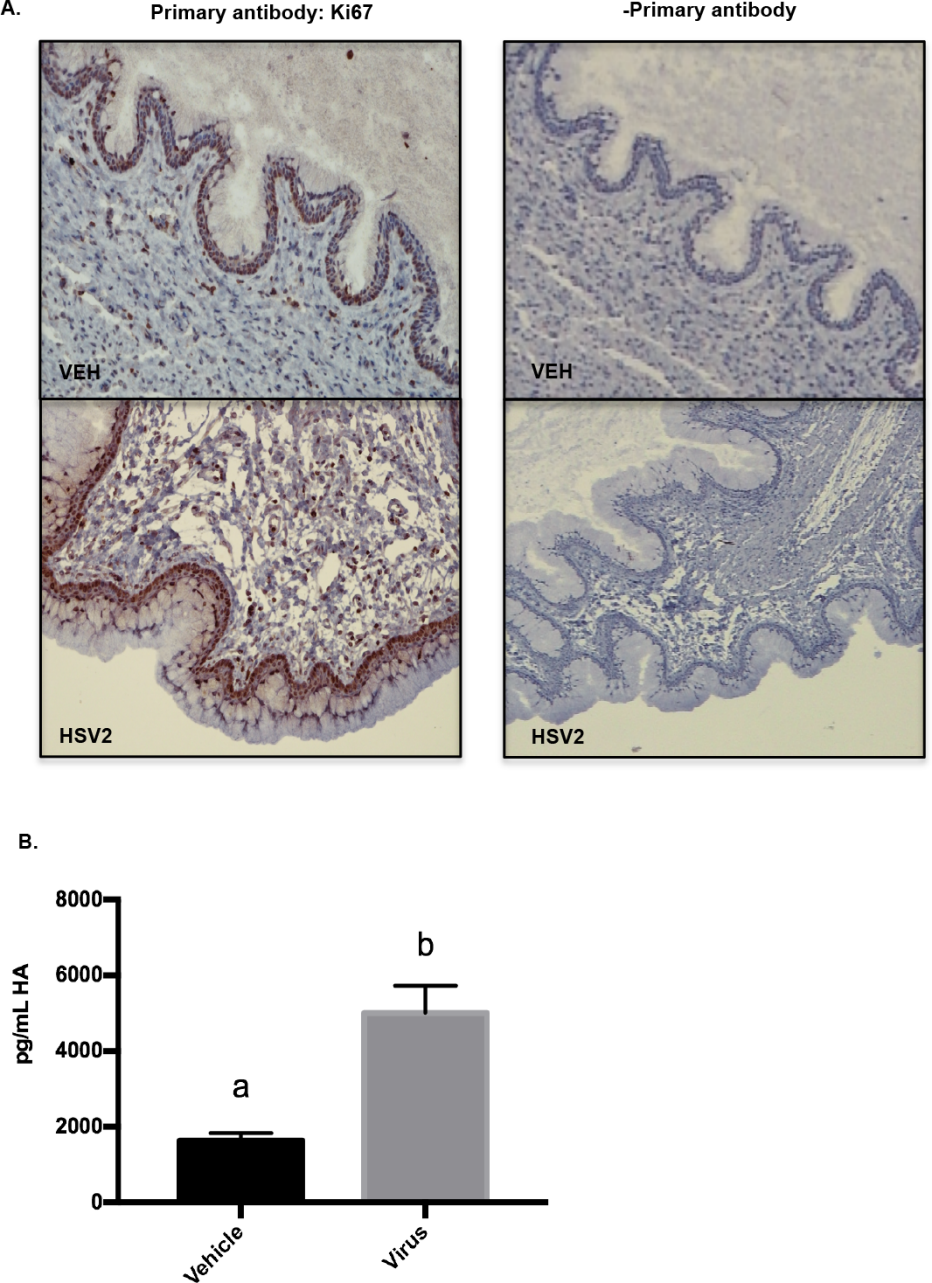
Epithelial cell proliferation and hyaluronic acid are increased by HSV2 infection of the pregnant cervix. (A) Cervical epithelial cell proliferation as determined by Ki-67 staining at GD15.5 in vehicle (VEH) and virus (HSV2) treated mice. There was no staining in control assay lacking primary antibody. (B) Hyaluronic acid was quantified in cervical flushes from vehicle and virus-treated animals at GD15.5 using ELISA (a<b, t-test, p = .012). Animal treatments: Vehicle, n = 8; Virus, n = 8.

Both epithelial cell proliferation and hyaluronic acid (HA) synthesis[27] increase during cervical ripening therefore we determined how they were affected by HSV2. At GD15.5, HSV2-infected mice had increased epithelial cell proliferation compared to GD15.5 NT mice, as determined by Ki-67 staining (Fig 4A). Hyaluronic acid was quantified in the cervical flushes from GD15.5 mice with and without viral infection and was significantly increased in flushes from the HSV2-infected mice (Fig 4B)(a<b, t-test, p = .012).

### Estrogen receptor-α and HA are increased by HSV2 in the human cervical epithelial cell line, ECT-1

**Fig 5:**
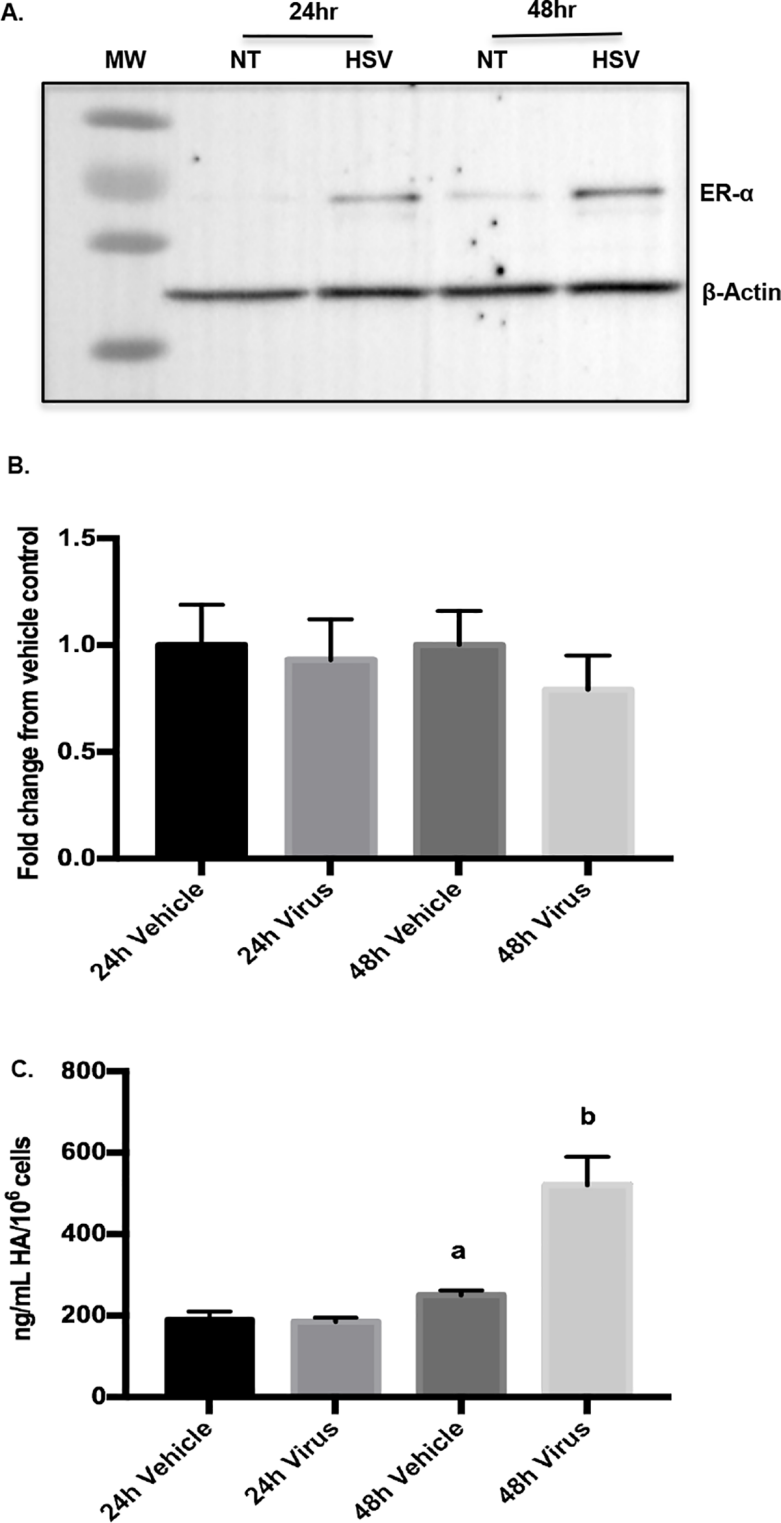
Estrogen receptor-alpha and HA are increased by HSV2 in the human cervical epithelial cell line, ECT-1. ECT-1 cells were treated with vehicle or HSV2 for 24h or 48h. (A) Representative Western blot analysis of ER-alpha, and actin loading control. (B) Quantitative PCR was used to analyze ERA mRNA expression in ECT-1 with and without HSV2 (ANOVA, n.s.). (C) Hyaluronic acid was quantified in ECT-1 conditioned medium 24 or 48h after treatment with vehicle or HSV-2 using ELISA (24h Veh vs Virus: t-test, n.s.; 48h Veh vs Virus: a<b, t-test, p = .017). Figures represent results of 4 independent in vitro experiments.

Next, to investigate the mechanism of HSV2-associated changes in hormone responsiveness, we utilized the human epithelial cell line, ECT-1. First, we determined if ECT-1 cells had a response to HSV2 that was similar to the mouse cervical epithelium. While HSV2 did increase ER-alpha protein (Fig 5A), infection did not change *ESR1* mRNA (Fig 5B)(analysis of variance (ANOVA), n.s.), suggesting protein stabilization as opposed to increased transcription. We also determined that HSV2 increased HA in ECT-1, as it did *in vivo*. Cells were infected with HSV2 (10^5^ PFU) and ECT-conditioned media was collected at 24h and 48h post-infection. At 48h, HA was increased in the media of HSV2 infected cells, compared to the NT controls (Fig 5C) (24h NT vs HSV2: t-test, n.s.; 48h NT vs HSV2: a<b, t-test, p = .017).

### Herpes simplex virus-2 regulates ER-α and HA via Src kinase in ECT-1 cells

**Fig 6:**
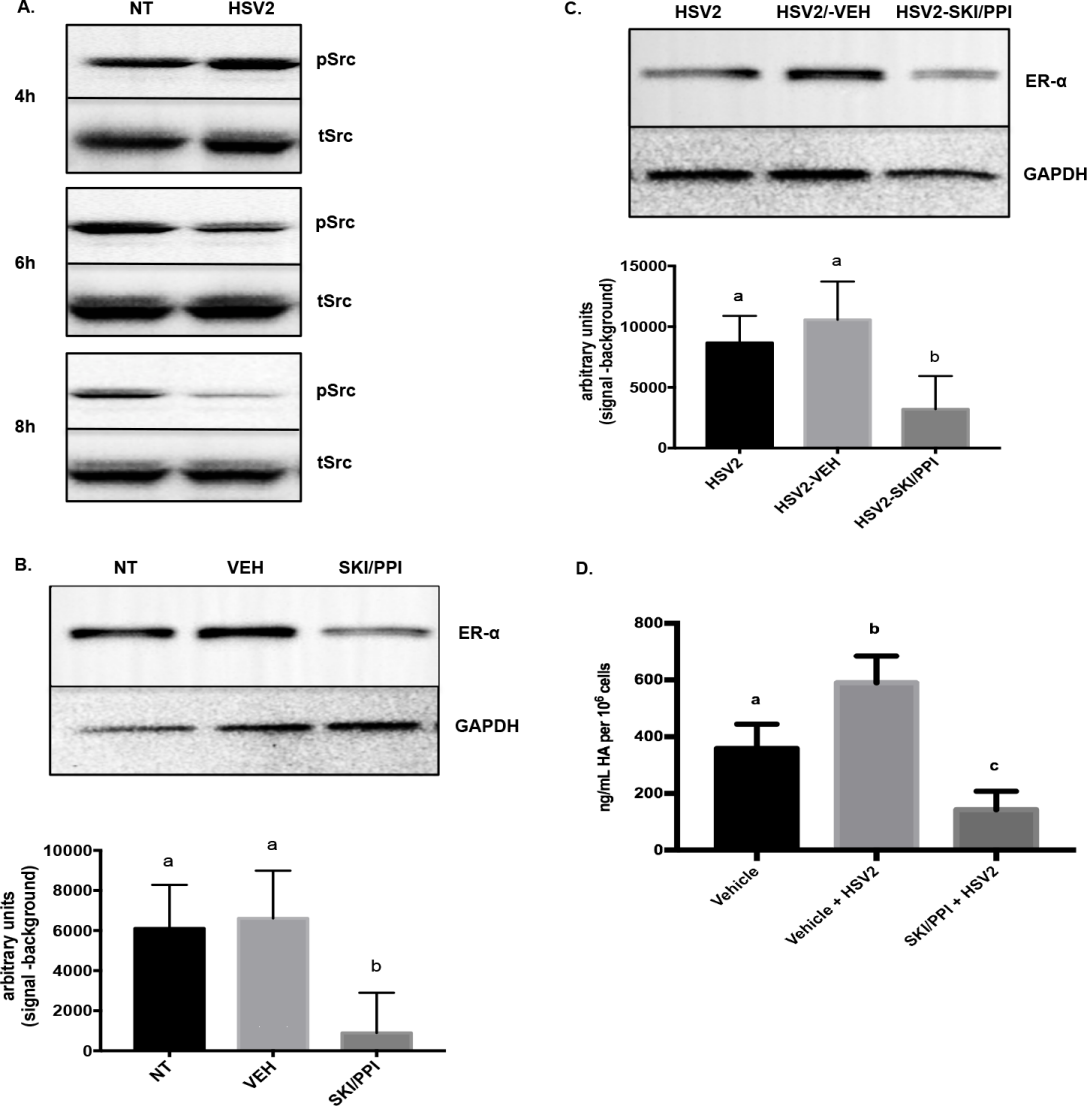
Herpes simplex virus-2 regulates ER-alpha and HA via Src kinase in ECT-1 cells. (A) Src kinase activation was characterized in ECT-1 cells 4, 6, and 8h post-infection with HSV2 using Western blot analysis with an antibody specific for Src Tyr527 phosphorylation (pSrc) or total Src (tSrc). (B) ECT-1 cells were treated with vehicle (DMSO) or Src inhibitor (SKI/PP1). ER-alpha was quantified using densitometry following Western blot analysis (ANOVA with Tukey’s multiple comparison; NT vs VEH, n.s.; NT vs SKI/PPI, p< .02; VEH vs SKI/PPI, p< .01). (C) ECT-1 cells were treated with HSV2, HSV2 and vehicle (DMSO), or HSV2 and Src kinase inhibitor, and ER-alpha was quantified using densitometry following Western blot analysis (ANOVA with Tukey’s multiple comparison; HSV2 vs VEH/HSV2, n.s.; HSV2 vs SKI/PPI-HSV2, n.s.; VEH/HSV2 vs SKI/PPI-HSV2, p< .04) (D). ECT-1 were treated with vehicle (DMSO), HSV2 and vehicle (DMSO), or HSV2 and Src kinase inhibitor, and HA was quantified in the conditioned medium using ELISA (ANOVA with Tukey’s multiple comparison; a<b, p = .012; a>c, p = .026; b>c, p = .0003). Figures represent results of 4 independent in vitro experiments.

We then used ECT-1 cells to determine how HSV2 stabilized ER-alpha protein and induced changes in HA expression. Since herpesviruses interact with integrins on the cell surface[28] we postulated integrin signaling might lead to kinase activation, which could affect ER-alpha stabilization. We tested the response of several focal adhesion kinases to HSV2 infection and determined the tyrosine kinase, Src, was affected by infection. ECT-1 cells were infected with HSV2 (10^5^ PFU) and Src-Tyr527 phosphorylation was quantified at multiple time points from 30m up to 8h post-infection. This phosphorylation site is an inhibitor of function and acts as the primary regulator of Src, therefore, loss of phosphorylation at Tyr527 indicates Src is active. We found that viral infection reduced phosphorylation (i.e. activated Src) at 6h and 8h post-infection (Fig 6A, S1 Fig). To determine if Src could affect ER-alpha, ECT-1 cells were treated with Src inhibitor, SKI/PP1, which resulted in decreased ER-alpha protein (Fig 6B, S1 Fig) (ANOVA with Tukey’s multiple comparison; NT vs VEH, n.s.; NT vs SKI/PPI, p< .02; VEH vs SKI/PPI, p< .01). Finally, to test if HSV-associated changes in ER-alpha were mediated by Src kinase, we inhibited Src (SKI-PP1) prior to HSV2 infection and again measured ER-alpha protein. When ECT-1 cells were treated with the Src inhibitor, HSV2 infection no longer caused an increase in ER-alpha protein (Fig 6C, S1 Fig) suggesting HSV2 mediates ER-alpha protein stability by activating Src kinase in cervical epithelial cells (ANOVA with Tukey’s multiple comparison; HSV2 vs VEH/HSV2, n.s.; HSV2 vs SKI/PPI-HSV2, n.s.; VEH/HSV2 vs SKI/PPI-HSV2, p< .04). Finally, we tested if Src mediated the HSV-associated changes in HA. When we infected ECT-1 with HSV2 in the presence of Src inhibitors (SKI-PP1), the virus-associated increase in HA was diminished (Fig 6D), (ANOVA; Tukey’s multiple comparison; a<b, p = .012; a>c, p = .026; b>c, p = .0003).

## Discussion

For the first time, to our knowledge, we report that intra-vaginal HSV2 infection increases rates of preterm birth following intra-vaginal bacterial infection in a mouse model of pregnancy. Specifically, HSV2 causes significant remodeling of collagen in the cervical stroma, increased expression of ER-alpha and PR in the cervical epithelium, increased epithelial cell proliferation, and up-regulated HA synthesis and/or shedding. Furthermore, using ECT-1 cells, we also discovered that HSV2 activates Src kinase, which mediates the increase in ER-alpha and HA. These results demonstrate that viral infection of the cervix, specifically, can significantly change cervical structure and function during pregnancy and these changes are associated with reduced protection against bacterial infections and PTB.

One intriguing finding of this study was the aberrant expression of ER and PR in cervical epithelial cells of HSV2-infected mice. Cervical ripening at term coincides with a switch from progesterone (P_4_) to estrogen (E_2_) dominance within the cervical tissue. In rodent models, treatment with a PR antagonist is sufficient to induce cervical ripening and in women, intra-vaginal P_4_ can delay cervical ripening and labor. While considerably less is known about the specific role of E_2_, loss of P_4_ following ovariectomy in rats did not induce ripening unless E_2_ concentrations were maintained, suggesting E_2_ also has an active role [29]. The shift in hormone dominance within the cervix at term is attributed to changes in local hormone metabolism and changes in receptor expression [22, 23, 30]. Therefore, it is possible that HSV2-associated expression of ER in cervical epithelial cells increases tissue sensitivity to E_2_, which could mediate the cervical changes we observe. We postulate that, in vivo, E_2_ ligand binds ER and is also responsible for induction of PR in epithelial cells. Interestingly, HSV2 infection led to increased ER in ECT-1 (via protein stabilization, not transcription), but PR mRNA and protein were not affected. It is likely PR was not up-regulated in vitro because we did not add E_2_ ligand to our culture.

Another phenotype associated with cervical HSV2 was a dramatic reorganization of the cervical stroma, including reduced cell density and loosening of the collagen fiber network. Although it is still unclear how these changes are mediated, this phenotype could be triggered by changes in hormone signaling, or by changes in immune cell populations or local immune cell functions[31]. We speculate that viral infection affects the cervix by increasing local E_2_-sensitivity that could affect factors that regulate collagen solubility and crosslinking. The virus could also be affecting cervix structure by potentiating the activation of immune cells, thus activating MMPs that degrade the collagen network[9]. These questions are the subject of continued investigation by our laboratory and others.

We also observed an increase in cervical HA associated with HSV2 infection. Hyaluronic acid synthesis has been associated with hormone-, and infection-, induced cervical ripening, a result of increased expression of HA synthase-2 or -3 (*Has2/3*). Interestingly, *Has2* is positively regulated by estradiol in the cervix[27]. This is relevant to our study because HA synthesis was increased in HSV2 infected cervices, which had increased expression of ER-alpha. Unfortunately, we could not use the ECT-1 to determine the role of ER-alpha signaling in HSV-induced HA synthesis because cervical epithelial cells do not properly respond to E_2_ without the stroma. However, we did determine that ER-alpha stabilization, and HA synthesis, were dependent on Src kinase activation following viral infection. Therefore, Src kinase could be an important mediator of cervical changes, such as increased HA, that are associated with HSV2 infection. These changes in HA could affect the biochemical makeup of the stroma and/or affect the mucus barrier. These functions are dependent on the form of HA that is upregulated, long or short chain, but this was not determined in this study.

**Fig 7:**
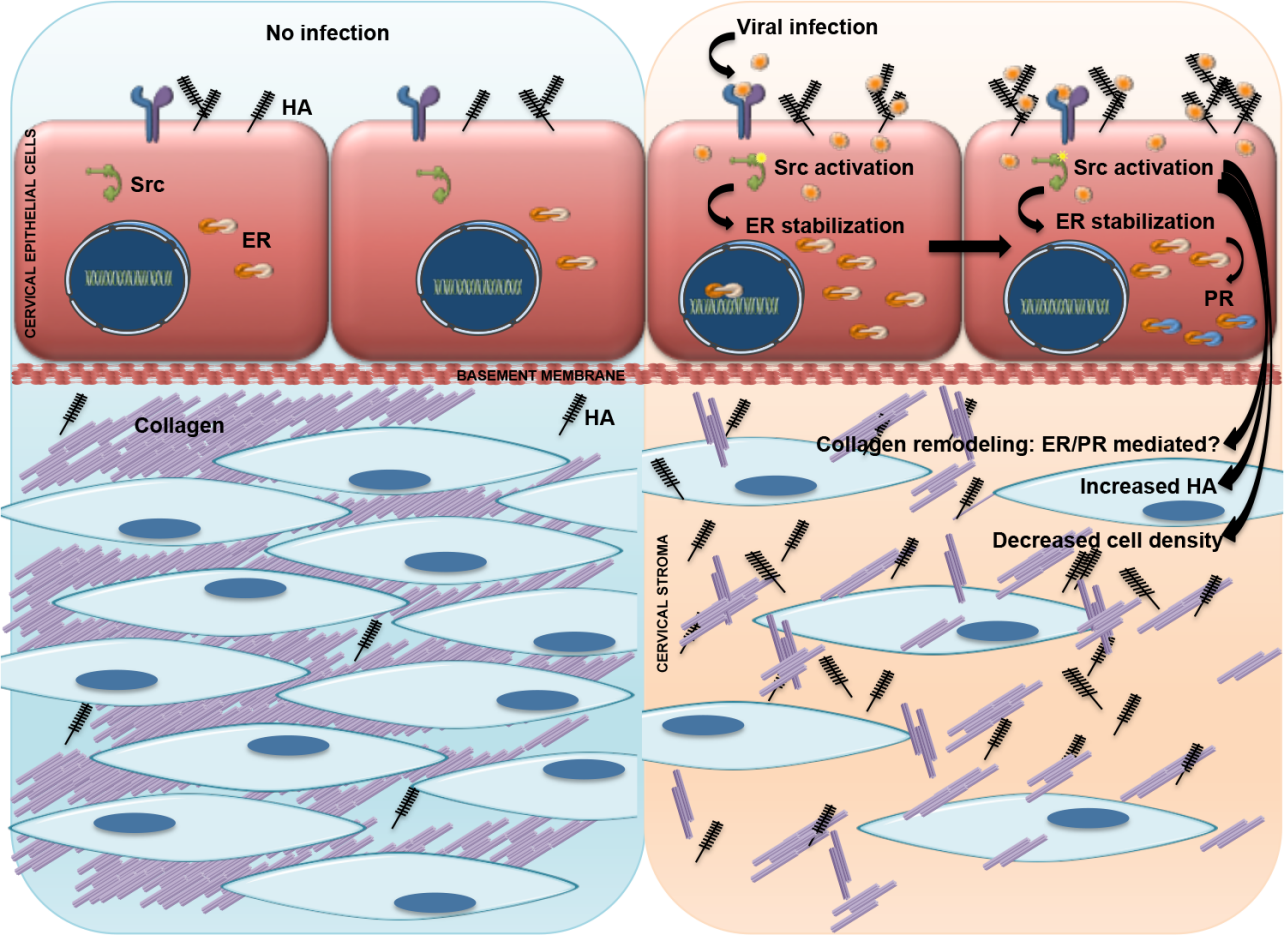
Model. We propose a model where virus-associated activation of Src kinase results in stabilization of ER-alpha leading to increased expression of PR and increased HA synthesis in the epithelium. Furthermore, we suggest these changes in hormone sensitivity affect the structure of the cervical stroma by affecting collagen solubility and/or crosslinking.

In conclusion, we propose a model where virus-associated activation of Src kinase results in stabilization of ER-alpha leading to increased expression of PR, and increased HA synthesis in the epithelium. Furthermore, we suggest these changes in hormone sensitivity affect the structure of the cervical stroma by affecting collagen organization (Fig 7). These changes are mediated by changes in collagen solubility and activation of local immune cells, all under the influence of sex hormones. These changes, mimicking those of cervical ripening, affect the structure and barrier function of the cervix, potentially leading to increased access to ascending infections that trigger PTB. Ongoing studies are investigating the specific molecular and structural changes in the cervical barrier associated with this phenotype. Studies like this are necessary to understand how cervical viral infections increase the risk for PTB in women. Based on these results, we could predict that HSV2 infection might weaken the cervix or even induce premature cervical ripening in some women. It is our hope that understanding the mechanism of virus-induced cervical remodeling will help us provide targets for clinical intervention.

## Supporting information

S1 Fig Uncut Western blots. Uncut blots associated with Fig 6A, Fig 6B and Fig 6C.
(TIFF)
