# Correction: Cervical HSV-2 infection causes cervical remodeling and increases risk for ascending infection and preterm birth

**DOI:** 10.1371/journal.pone.0199566

**Published:** 2018-06-18

**Authors:** Devin McGee, Arianna Smith, Sharra Poncil, Amanda Patterson, Alison I. Bernstein, Karen Racicot

Two references were omitted from the seventh sentence under the subheading “Histology” in the Methods section.

The sentence should read: For collagen staining, 10μm tissue sections were stained with picrosirius red (Polysciences Inc, Warrington, PA) as previously described (Junqueira et al., 1980; Yu et al., 1995).

The references are:

Junqueira L, Zugaib M, Montes G, Toledo O, Krisztán R, Shigihara K. Morphologic and histochemical evidence for the occurrence of collagenolysis and for the role of neutrophilic polymorphonuclear leukocytes during cervical dilation. American Journal of Obstetrics and Gynecology. 1980;138(3):273–81.

Yu SY, Tozzi CA, Babiarz J, Leppert PC. Collagen Changes in Rat Cervix in Pregnancy—Polarized Light Microscopic and Electron Microscopic Studies. Experimental Biology and Medicine. 1995Jan;209(4):360–8.

One reference was omitted from the tenth sentence under the subheading “Histology” in the Methods section.

The sentence should read: Specifically, after photomicrographs were converted to gray scale, they were inverted and OD was calculated using a calibrated threshold and the Rodbard standard curve (NIH Image J software) as previously described (Kirby et al., 2005).

The reference is: Kirby LS, Kirby MA, Warren JW, Tran LT, Yellon SM. Increased Innervation and Ripening of the Prepartum Murine Cervix. Journal of the Society for Gynecologic Investigation. 2005;12(8):578–85.
