# Supplementary figures and images for: Cervical HSV-2 infection causes cervical remodeling and increases risk for ascending infection and preterm birth
Source: PLoS One. 2017 Nov 30;12(11):e0188645. doi: 10.1371/journal.pone.0188645 (PMC5708831; doi:10.1371/journal.pone.0188645)

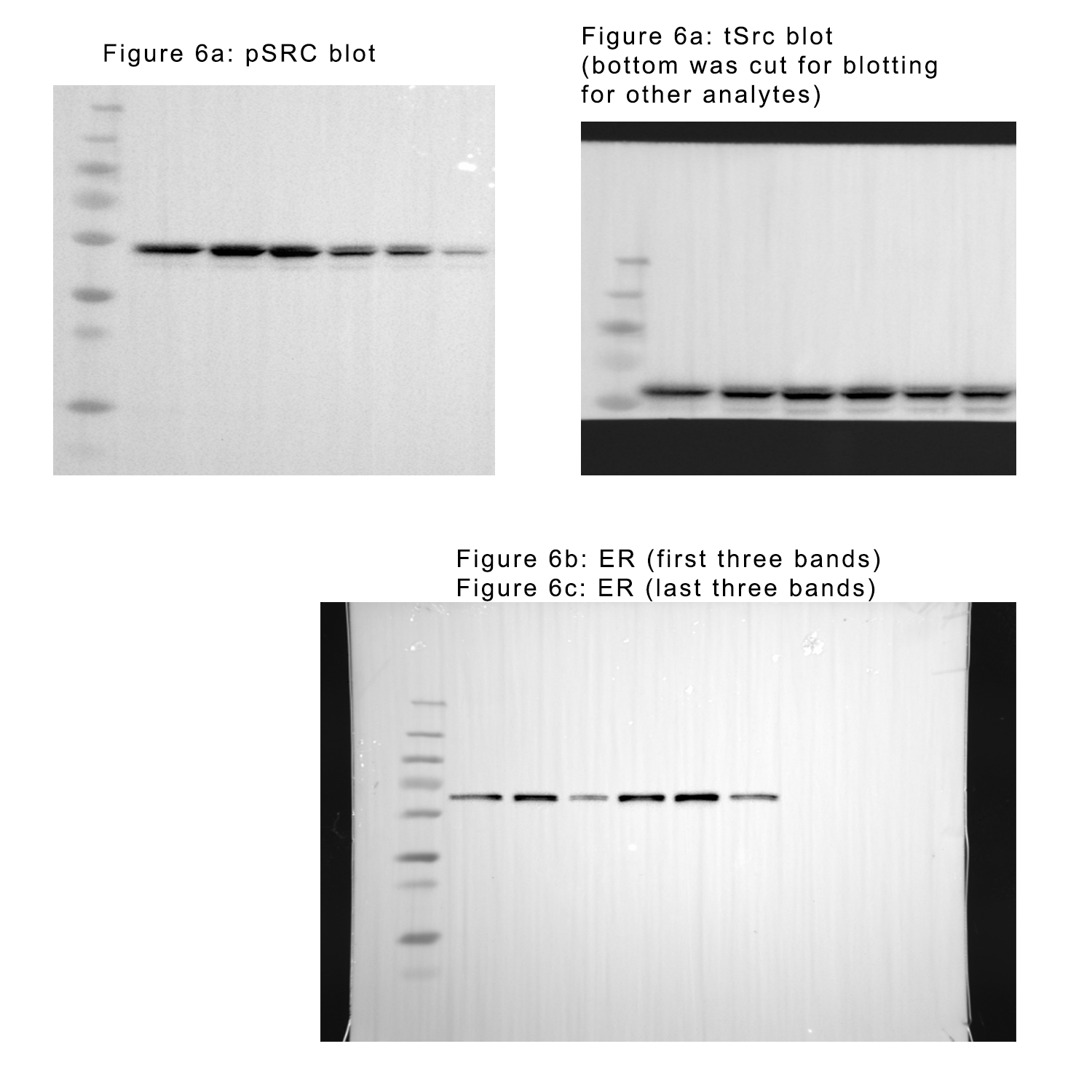

Supplement: S1 Fig — Uncut blots associated with Fig 6A, Fig 6B and Fig 6C. (TIFF) [file pone.0188645.s001.tiff]
